# Female Gender and Acne Disease Are Jointly and Independently Associated with the Risk of Major Depression and Suicide: A National Population-Based Study

**DOI:** 10.1155/2014/504279

**Published:** 2014-02-11

**Authors:** Yi-Chien Yang, Hung-Pin Tu, Chien-Hui Hong, Wei-Chao Chang, Hung-Chun Fu, Ji-Chen Ho, Wei-Pin Chang, Hung-Yi Chuang, Chih-Hung Lee

**Affiliations:** ^1^Department of Dermatology, Kaohsiung Chang Gung Memorial Hospital and Chang Gung University College of Medicine, Kaohsiung 83301, Taiwan; ^2^Graduate Institute of Clinical Medical Sciences, Chang Gung University, College of Medicine, Taoyuan 33302, Taiwan; ^3^Department of Public Health and Environmental Medicine, School of Medicine, College of Medicine, Kaohsiung Medical University, Kaohsiung 80756, Taiwan; ^4^Department of Dermatology, Kaohsiung Veterans General Hospital and National Yang Ming University Hospital, Kaohsiung 81362, Taiwan; ^5^Master Program for Clinical Pharmacogenomics and Pharmacoproteomics, School of Pharmacy, Taipei Medical University, Taipei 10478, Taiwan; ^6^Department of Clinical Pharmacy, School of Pharmacy, Taipei Medical University, Taipei 10478, Taiwan; ^7^Department of Obstetrics and Gynecology, Kaohsiung Chang Gung Memorial Hospital and Chang Gung University College of Medicine, Kaohsiung, Taiwan; ^8^Department of Healthcare Management, Yuanpei University, Hsinchu 30015, Taiwan; ^9^Department of Environmental and Occupational Medicine, Kaohsiung Medical University Hospital, Kaohsiung 80756, Taiwan; ^10^Department of Public Health, Kaohsiung Medical University, Kaohsiung 80756, Taiwan

## Abstract

Acne is a common disease in adolescence with female preponderance. It could cause poor self-esteem and social phobia. Previous studies based on questionnaires from several thousands of adolescents showed that acne is associated with major depression and suicide. However, the gender- and age-specific risk of depression and suicide in patients with acne remain largely unknown. Using a database from the National Health Insurance, which included 98% of the population of Taiwan in 2006, we identified patients of acne, major depression, and suicide based on ICD-9-CM codes. Totally 47111 patients with acne were identified (16568 males and 30543 females) from 1 million subjects. The youths of 7–12 years had the highest prevalence of acne (14.39%). Major depression was more common in those with acne (0.77%) than controls (0.56% , *P* < 0.0001) regardless of gender. Multiple logistic regression showed an increased risk of major depression in women without acne (OR = 1.85, 95% CI 1.75–1.96). The risk is additive in women with acne (OR = 2.78, 95% CI 2.43–3.17). Similar additive risk of suicide was noticed in women with acne. In conclusion, acne and gender, independently and jointly, are associated with major depression and suicide. Special medical support should be warranted in females with acne for the risk of major depression and suicide.

## 1. Introduction

Acne is a common skin disease in adolescence and can persist, in some cases, into adulthood [1, 2]. The facial appearance is important in social interaction, self-image, and self-esteem. Although acne is not a life-threatening disease, it could be distressing and cause adverse psychosocial consequences such as depression, poor self-esteem, and social phobia in the patients.

Major depression is recognized as a persistent low mood accompanied by low self-esteem, feelings of worthlessness, and a loss of general interest. Suicide attempt is an indicator of emotional distress and tends to occur in patients with major depression. Major depression is a well-known major risk factor of suicide [3]. In Taiwan, suicide is a significant social problem as it is the third leading cause of death among the adolescent to the middle aged people [4].

As compared to men, in general, women have a higher chance of developing major depression, anxiety, and neurotic disorder and might have overall impaired quality of life [5]. Limited to the patients with acne, there is an increase of depressive symptoms in adolescents with acne [6–8]. In addition, several recent studies based on questionnaire survey have confirmed the association of acne with suicide attempts [7, 9] and body dysmorphic disorder [10]. In fact, an effective treatment of acne was accompanied by an improvement in self-esteem and mood [11].

Acne tends to affect females with a male to female ratio about 1/1.1–1.25 in Asians [12, 13]. We previously reported the epidemiological characteristics of acne and its associated disease burden in schoolchildren in Taiwan. Consistent with previous studies, more females are affected than males in our studies (M : F = 1/1.5–1.9) [14, 15]. In women, a high proportion of these acne cases are late onset [1, 2]. Although previous studies have found that acne is associated with the development of major depression and suicide, its impacts on the major depression and suicide may vary in people with different age, gender, and ethnic and cultural background. For example, most of the previous studies were limited to the adolescent populations. Large-scale studies regarding the association of acne and major depression and suicide from the childhood to adulthood are scarce. Moreover, although major depression and suicide tend to affect women and patients with acne, women are in fact more likely than men to develop acne. A question remains how acne by itself would be associated with the development of major depression and suicide. This makes it relevant to study the independent association between acne and major depression/suicide, as adjusted by genders and ages in a large population.

In this study, we aimed to determinate the age- and gender-specific prevalence of acne in Taiwan. In addition, we also evaluated the associations between acne and depressions and suicides. We also estimated the impact of female gender and acne disease on the risk of major depression and suicide.

## 2. Materials and Methods 

### 2.1. Patient Ascertainment

This is a population-based study using the claim data collected by the National Health Insurance (NHI). Taiwan NHI is a program of health-care system. In 2006, more than 98% of Taiwan's population (about 23 million) was enrolled in this program. Therefore, our study population consists of almost the whole population of Taiwan in 2006. Based on the NHI database of year 2006, we collected and analyzed data related to acne, major depression, and suicide attempt as defined by the International Classifications of Diseases, Ninth Revision, Clinical Modification (ICD-9-CM) codes. Ascertainment of acne involves the selection of ICD-9-CM codes for acne including 706.1 with other acne, 706.2 with sebaceous cyst, 706.3 with seborrhea, 706.8 with other specified diseases of sebaceous glands, and 706.9 with unspecified disease of sebaceous glands. Further, we chose ICD-9-CM codes for major depression, including 296.2 with major depressive disorder single episode and 296.3 with major depressive disorder recurrent episode. ICD-9-CM codes for suicide attempt include the following: E950 with suicide and self-inflicted poisoning by solid or liquid substances, E951 with suicide and self-inflicted poisoning by gases in domestic use, E952 with suicide and self-inflicted poisoning by other gases and vapors, E953 with suicide and self-inflicted injury by hanging strangulation and suffocation, E954 with suicide and self-inflicted injury by submersion (drowning), E955 with suicide and self-inflicted injury by firearms air guns and explosives, E956 with suicide and self-inflicted injury by cutting and piercing instrument, E957 with suicide and self-inflicted injuries by jumping from high place, E958 with suicide and self-inflicted injury by other and unspecified means, and E959 with late effects of self-inflicted injury.

### 2.2. Statistics

The prevalence of acne and depression was stratified by sex and age group. The 95% CI of the prevalence was estimated by the Poisson distribution model. In the univariate analysis, the association between acne and the prevalence of major depression and suicide was tested using Fisher's exact test for categorical variables. The relationship between acne and prevalence of the major depression was assessed by logistic regression. Results are presented as odds ratios (OR) and 95% confidence intervals (95% CI). Interactions between acne and gender were tested under multiple logistic regression models using an added interaction term (acne × gender) and main covariates, age categories. A *P* value of <0.05 was considered significant. All statistical analyses were performed by using the software packages SAS, version 9.3 (SAS Institute Inc., Cary, North Carolina, USA).

## 3. Results

### 3.1. Age- and Gender-Specific Prevalence of Acne in Taiwan

To investigate the general prevalence rate of acne in Taiwan, we used the Taiwan National Health Insurance (NHI) database with a random sampling of one million patients visiting health care providers in 2006 to estimate the overall prevalence of acne (Table 1). Among these one million subjects, totally 47111 patients with acne (ICD-9-CM 706.x except 706.0) from the one million subjects at risk were identified (including 16568 male patients and 30543 female patients). Our result showed that overall prevalence of acne in Taiwan in 2006 was 4.71% (95% CI, 4.67%–4.75%). People aged 7–12 years (elementary school age) had the highest risk of developing acne, with a prevalence rate at 14.39% (95% CI, 14.15%–14.63%). The prevalence decreased with age thereafter, but there were a significant number of patients experiencing acne after 19 years old, especially in females aged 19–42 years. Acne was relatively uncommon in people older than 42 years of age. To determine whether women were prone to acne, we further stratified the prevalence of acne by gender. The prevalence was higher in women, at a rate of 6.06% (95% CI, 5.99%–6.12%), than in men, at a rate of 3.34 (95% CI, 3.29%–3.39%). Male to female ratio of prevalence is around 1 : 1.81. Girls aged 7–12 years had the highest prevalence rate of acne (17.78%, 95% CI 17.42%–18.14%). In addition, in each age category, women were at higher risk than men of developing acne. In contrast to the decreasing trend of prevalence in men after the age of 13, women at high school age (age 13–18) remained at a higher risk of developing acne, reaching a prevalence at 14.15% (95% CI, 13.85%–14.46%).

### 3.2. Patients with Acne Tend to Develop Major Depression and Commit Suicide in Both Genders

To further define the impact of gender in the development of major depression and suicide attempt in patients with acne, we first performed the association analysis of acne and major depression/suicide as stratified by the gender. The overall prevalence of major depression and suicide attempts in patients with acne was 0.77% and 0.01%, respectively (Table 2). The result demonstrated that major depression (0.77%) was more common in those with acne than controls (0.56%, *P* < 0.0001). We also observed an increased risk of suicide among patients with acne although it did not reach a statistical significance. In general population, the prevalence of major depressions is more common in women than those in men at about 1.5-fold [5]. Concurrently, the prevalence of major depression and that of suicide are both higher in women with acne than those in men with acne. Taken together, our data confirmed that patients with acne tend to develop major depression in both genders and suggested that genders might affect the risk of developing major depression in patients with acne.

### 3.3. Acne and Gender, Independently and Jointly, Are Associated with the Risk of Major Depression and Suicide

We have shown that patients with acne and females are both associated with an increased risk of major depression and suicide (Table 2) as adjusted by age with a multiple logistic regression model. To further determine whether gender and acne both contributed to the risk of major depression, we performed further multiple logistic regression models using an added interaction term (acne × gender) and main covariates as age categories (Table 3). Our results showed that both acne and women are associated with the increased risk of major depressions. Without acne, women have an odds ratio of 1.85 (95% CI 1.75–1.96) to develop major depression as compared to men. In men, men with acne have a 2.12-fold (95% CI 1.73–2.60) increased risk of developing major depression than men without. The effects of women and acne in development of major depressions are additive since women with acne have a 2.78-fold (95% CI 2.43–3.17) increased risk of developing major depression (1.85 + 2.12-1~2.78).

Similarly, our results showed that both acne and women are associated with the risk of suicide (Table 4). Without acne, women have an odds ratio of 1.96 (95% CI 1.18–3.23) to commit suicide as compared to men. In contrast, men with acne do not have an increased risk of suicide than men without (OR = 1.01, 95% CI 0.15–8.24). In patients with acne, however, women with acne showed an overall increase of risk of suicide by 3.17-fold (95% CI 1.27–7.94). Special attention should be paid to the female patients with acne in terms of their particular risk of committing suicide.

## 4. Discussions

This study analyzed data from a large random sample of the general population in Taiwan. Acne was commonly thought to be only a skin problem that usually affected the adolescence. This study indicated the 7–12-year-old children being affected at the higher rates than adolescents (13–18 years old) and many adults also experience acne. In addition, females were more vulnerable to acne than males in all age groups. Consistent with our previous community-based study conducted by dermatologist's direct inspection and diagnosis [15], the trend of female predominance in this age group was observed. However, the prevalence of acne in school children was higher (M/F = 12.3%/23.4%) in the previous study [15] than that in the current study (M/F = 10.78%/17.78%). This might result from the fact that not all the patients with acne seek medical help. The current study only included patients who had sought treatment for their acne. Therefore it may have underestimated the total prevalence of acne in the general population. Interestingly, based on these two studies with different experimental setup, prevalence of acne in males is similar (12.3% versus 10.78%), but a subtle difference existed in females (23.4% versus 17.78%). Therefore, the dermatologists and pediatricians should provide earlier medical, educational intervention to these young patients and their parents to improve the awareness of the management of acne and prevent the sequelae such as permanent scarring and the comorbid psychosocial illness.

Clearance of acne was generally expected to occur spontaneously in early adulthood. However, acne remains a common skin disease after the second decade [1, 16, 17]. Our results also demonstrated that acne lesion can persist into middle age for both genders. Females were at higher rate of 2-fold greater than males. This is consistent with the findings of female preponderance from a previous study in adult acne based on the participant's own perception of acne [1]. The current study and studies from others all found that females with acne were positively correlated with the psychological damages [18–20]. Thus, assessing psychological distress among the women with adulthood acne would be warranted.

We found that major depression was more prevalent by 2-fold in patients with acne (0.77%) than general population in Taiwan (0.35%) [5]. Furthermore, the risk of major depression was significantly higher in people with acne than those without (*P* < 0.0001). Previous studies have reported that acne could lead to psychological problems and it affects more females than males [8, 13, 18, 21, 22]. Similar impacts of acne on psychological impairment in both genders have been also demonstrated [9]. Our results indicate that the female gender and acne are two independently contributory factors to developing major depression.

The significance of the association of acne and major depression was higher in males and there was 2.12-fold increased risk of developing major depression in males with acne than those without. This claim data did not include disease severity as an outcome. Since the disease severity of acne is associated with social impairment [9], a possible explanation for the higher prevalence of major depression in females with acne but a more significant association of acne and major depression in males might be related to the higher severity of acne in school students aged 4–18 year old [23] and postadolescent [16].

Regarding the association of suicide and acne, an increased risk of suicide attempts in young people presenting with acne has been reported [7, 9, 24]. Although the association of acne and suicide did not reach statistical significance in this study, we observed the trend of increased risk of suicide among patients with acne. The impact of acne on different genders remains controversial. A questionnaire-based study among adolescents indicated that the impact of acne on suicidal ideation was equivalent in both genders [9]. However, among our male patients, the impact of acne on suicide was very limited, which was consistent with a previous study [25]. In this study, female gender and acne were two independent risk factors of suicide. Nevertheless, some authors have proposed mental distress as a possible cause of acne. Further studies focusing on the causal relationship of acne and these psychiatric problems remained to be investigated.

Isotretinoin is an effective therapeutic option for severe and recalcitrant acne. Use of isotretinoin was reported to be associated with depression and suicide attempts in patients with acne [26–28] although the direct casual effect in remains obscure. In the contrary, some recent studies showed that isotretinoin has favorable effects on depression and suicide attempts subsequently after successful treatment of acne [29–32]. The effect of isotretinoin on the depression and suicide becomes controversial. We tried to use the current study design to answer whether isotretinoin affected the development of major depression and suicide. However, from the NHI database, only 18 from 47111 patients with acne took isotretinoin. This underestimation of isotretinoin use resulted from the fact that isotretinoin reimbursement was limited to the patients with refractory nodular cystic acne based on the policy of NHI and the majority of the acne patients treated with isotretinoin paid out of their own pockets. Thus, we could not address whether the isotretinoin affected depression or suicide due to the limitation of the NHI database in this study.

Our study highlighted that acne is an early onset and chronic skin disease which may influence mental health throughout lifetime, especially in females. Acne could not be considered simply as a superficial problem in physical appearances. The requirement of active screen for the comorbidities of psychological diseases, such as major depression and suicide, among the acne patients should be warranted.

Although the numbers of patients with acne identified are tens of thousands, there are some intrinsic limitations using the NHI database. Firstly, the doctors may not give an accurate ICD-9-CM codes upon visit. Patients with different ages, genders, and diseases may have different thresholds to visit doctors. Nonetheless, in this study, we still observed consistent female to male ratios and prevalence in patients with acne as compared to our previous studies, suggesting that the analysis based on NHI database may reflect at large the de facto situation. Secondly, the NHI database and the insurance forms could not address whether the females seek medical help earlier or the females were affected by the acne earlier. However, our previous community-based study indicated that comedones were actually identified in girls at age 6, which was 1 year earlier than that in boys. Thirdly, we could not distinguish reactive or endogenous depressions using this database. Further hospital-based case control study focusing on the association of acne and reactive/endogenous depression is needed.

We concluded that female gender and acne disease are jointly and independently associated with the risk of major depression and suicide. The results may provide useful decision making information to the public health policy decision makers to estimate the need of appropriate health care for patients with acne in our community. Education and encouragement of the people with acne, women in particular, to seek an appropriate medical, mental, and social support not only improve the physical appearance and self-esteem but also decrease the associated social consequences and burdens.

## Figures and Tables

**Table 1:** Prevalence of acne in Taiwan, 2006.

	ICD-9-CM 706.x	At risk population	Prevalence (95% CI)
	*n*	*n*	
Men			
Overall	16568	495838	3.34% (3.29%–3.39%)
Age category, years			
0–6	4234	110114	3.85% (3.73%–3.96%)
7–12	4291	39800	10.78% (10.48%–11.09%)
13–18	2732	46295	5.90% (5.69%–6.12%)
19–24	1482	48014	3.09% (2.94%–3.25%)
25–30	956	51107	1.87% (1.76%–1.99%)
31–42	1355	96541	1.40% (1.33%–1.48%)
>42	1518	103967	1.46% (1.39%–1.54%)
Women			
Overall	30543	504162	6.06% (5.99%–6.12%)
Age category, years			
0–6	4711	100662	4.68% (4.55%–4.81%)
7–12	7549	42469	17.78% (17.42%–18.14%)
13–18	7098	50167	14.15% (13.85%–14.46%)
19–24	4184	51782	8.08% (7.85%–8.32%)
25–30	2957	54410	5.44% (5.25%–5.63%)
31–42	2859	98842	2.89% (2.79%–3.00%)
>42	1185	105830	1.12% (1.06%–1.19%)
Combined group			
Overall	47111	1000000	4.71% (4.67%–4.75%)
Age category, years			
0–6	8945	210776	4.24% (4.16%–4.33%)
7–12	11840	82269	14.39% (14.15%–14.63%)
13–18	9830	96462	10.19% (10.00%–10.38%)
19–24	5666	99796	5.68% (5.54%–5.82%)
25–30	3913	105517	3.71% (3.60%–3.82%)
31–42	4214	195383	2.16% (2.09%–2.22%)
>42	2703	209797	1.29% (1.24%–1.34%)

**Table 2:** Patients with acne tend to develop major depression and commit suicide in both genders.

	ICD-9-CM 706.x	Non-ICD-9-CM 706.x	*P*
	*n*	*n*	
*Men*			
Major depression (296.2 + 296.3)			
+	101 (0.61)	1851 (0.39)	<0.0001
−	16467 (99.39)	477419 (99.61)	
Suicide (E950–E959)*			
+	1 (0.01)	23 (0.00)	0.5577
−	16567 (99.99)	479247 (100.00)	
*Women *			
Major depression (296.2 + 296.3)			
+	261 (0.85)	3453 (0.73)	0.0142
−	30282 (99.15)	470166 (99.27)	
Suicide (E950–E959)*			
+	6 (0.02)	45 (0.01)	0.1281
−	30537 (99.98)	473574 (99.99)	
*Combined group *			
Major depression (296.2 + 296.3)			
+	362 (0.77)	5304 (0.56)	<0.0001
−	46749 (99.23)	947585 (99.44)	
Suicide (E950–E959)*			
+	7 (0.01)	68 (0.01)	0.0900
−	47104 (99.99)	952821 (99.99)	

*Fisher's exact test.

**Table 3:** Acne and gender, independently and jointly, associated with the risk of major depression.

	Major depression	Nonmajor depression	Adjusted OR (95% CI)*	*β*, *P* for interaction
Acne (no) and men	1851 (32.67)	477419 (48.01)	1.00	
Acne (no) and women	3453 (60.94)	470166 (47.28)	1.85 (1.75–1.96)	
Acne (yes) and men	101 (1.78)	16467 (1.66)	2.12 (1.73–2.60)	
Acne (yes) and women	261 (4.61)	30282 (3.05)	2.78 (2.43–3.17)	−0.35, 0.0043

*Adjusted age categories.

**Table 4:** Acne and gender, independently and jointly, associated with the risk of suicide.

	Suicide	Nonsuicide	Adjusted OR (95% CI)*	*β*, *P* for interaction
Acne (no) and men	23 (30.67)	479247 (47.93)	1.00	
Acne (no) and women	45 (60.0)	473574 (47.36)	1.96 (1.18–3.23)	
Acne (yes) and men	1 (1.33)	16567 (1.66)	1.10 (0.15–8.24)	
Acne (yes) and women	6 (8.00)	30537 (3.05)	3.17 (1.27–7.94)	0.39, 0.7280

*Adjusted age categories.
